# The Influence of Al and Nb on the Low Oxygen Pressure Pre-Oxidation Behavior of Fe-35Ni-20Cr-xAl-yNb Alloys at 1000 °C

**DOI:** 10.3390/ma17092086

**Published:** 2024-04-29

**Authors:** Lang Chen, Manman Yuan, Ya Liu, Junxiu Chen, Changjun Wu, Xuping Su

**Affiliations:** 1 School of Materials Science and Engineering, Changzhou University, Changzhou 213164, Chinayliu@cczu.edu.cn (Y.L.); czdxcjx@cczu.edu.cn (J.C.); wucj@cczu.edu.cn (C.W.); 2 Jiangsu Key Laboratory of Materials Surface Science and Technology, Changzhou University, Changzhou 213164, China; 3 Jiangsu Collaborative Innovation Center of Photovoltaic Science and Engineering, Changzhou University, Changzhou 213164, China

**Keywords:** Fe-Ni-Cr-based alloys, low oxygen pressure, pre-oxidation, oxidation behavior, phase diagram of oxidation

## Abstract

To investigate the impact of Al and Nb elements on the formation of a protective oxide layer on the surface of Fe-35Ni-20Cr-xAl-yNb (x = 0, 2, 4, 6 wt.%; y = 0, 1, 2 wt.%) alloys, their oxidation behavior was examined at 1000 °C, 10^−17^ atm. and 10^−25^ atm. oxygen pressure, and the oxidation mechanism was analyzed by Factsage and Pandat calculations. Enhancing the Al content at 10^−17^ atm. inhibited the generation of FeCr_2_O_4_ on the alloy surface and increased the Al content in the M_2_O_3_ layer. When the Al content exceeded 6 wt.%, the oxide film partially peeled off. It was found that the addition of Nb increased the activity of Cr and Al and decreased the activity of Ni and Fe and promoted the formation of Al_2_O_3_, and the appearance of Nb_2_O_5_ in the subsurface layer increased the density of the oxide film. In addition, under an oxygen pressure of 10^−25^ atm., the only protective layer on the surface of the alloy comprised of Al_2_O_3_. The experimental results demonstrated that the Fe-35Ni-20Cr-4Al-2Nb alloy generated a continuous and dense Al_2_O_3_ protective film, and the reduction in oxygen pressure and the addition of Nb elements were favorable for selective external oxidation of Al_2_O_3_.

## 1. Introduction

Cast heat-resistant austenitic stainless-steel Fe-Ni-Cr based alloy, as a high-temperature structural material, is widely used in high-temperature furnaces and refining processing equipment. Its high-temperature and high-carbon working environment places strict requirements on the oxidation resistance and carburization resistance requirements for equipment materials [1,2]. Stainless steel and Fe-Ni-Cr centrifugal casting alloys are commonly used, with their oxidation resistance effectively enhanced by the rapid formation of a surface Cr_2_O_3_ oxygen barrier layer [3], which obstructs the flow of oxygen into the substrate, and the formation of a low-oxygen site at the oxide–metal interface, thus reducing the solubility of O [4,5]. If the working temperature of the material is excessively high, Cr_2_O_3_ will continue to oxidize to form volatile CrO_3_ [6,7]. Moreover, in damp settings and under high gas flow rates [8,9], the Cr component in the substrate’s surface layer will deplete. If the Cr becomes depleted, the material will lose its antioxidant capabilities.

Aluminum oxide presents a lower growth rate and superior thermal stability compared to the chromium oxide film formed on the surface of conventional austenitic stainless steels [10,11]. Following comparison in high-temperature carbon and water vapor environments, the Al_2_O_3_ film shows improved stability when compared to Cr_2_O_3_ [12,13,14]. For instance, the 60HTR material developed by Henrik Asteman et al. relies on the formation of a stable and well-adhered Al_2_O_3_ protective film on the surface of the alloy to improve the high temperature corrosion resistance [15]. The addition of Al elements to Fe–Ni–Cr-based alloys and the production of a protective Al_2_O_3_ film on the surface has been studied; these types of alloy are referred to as AFA alloys (Aluminum-Forming Austenitic stainless steels) [16].

Generally, in low Al-content alloys, Al_2_O_3_ forms mainly in the internal oxidation zone, and the presence of Cr has a significant positive impact on Al_2_O_3_ film formation [17]. With the addition of Cr to the alloy, the rapidly formed Cr_2_O_3_ acts as a barrier layer to reduce the oxygen pressure between the substrate and oxide film interface and promotes the formation of internal Al_2_O_3_. High oxygen potential difference promotes outward diffusion of the Al element. In addition, Cr can reduce the critical content of Al in the alloy for the formation of Al_2_O_3_ outer oxide layer (third element effect) [18], which is favorable to the formation of Al_2_O_3_ film. The target alloy in this study is Fe-35Ni-20Cr-xAl-yNb (x = 0, 2, 4, 6 wt.%; y = 0, 1, 2 wt.%). In order to oxidize the exterior of the alloy to form a continuous and dense Al_2_O_3_ film, this can be achieved by increasing the Al elemental content of the alloy, but it is necessary to avoid an excessively high Al content, as this can lead to brittle alloys and poor formability [19]. Therefore, there exists an optimum concentration of Al content and its investigation deserves further research.

Brady et al. found that the increase in Nb and Al content were both conducive to the establishment and maintenance of protective Al_2_O_3_ fouling in the AFA alloy studied; they speculated that the increase in Nb content may reduce the oxygen solubility of the alloy [20,21], but that lower Al and Nb content is not conducive to the formation of an Al_2_O_3_ external oxide layer [21]. Initial efforts were made to increase the upper-temperature oxidation limit for the formation of Al_2_O_3_ in AFA steels by increasing the Nb content of AFA steels. The studies showed that Fe-20Ni-14Cr-3Al-1.5Nb-0.1C and Fe-20Ni-14Cr-3Al-2.5Nb-0.08C steels with 1.5 wt.% and 2.5 wt.% Nb additions exhibited excellent oxidation resistance in air at 1173 K [21,22]. This was attributed to the fact that Nb promotes the formation of an AlNi phase and the continuous formation of Al_2_O_3_ layers [3,13,16]. If more Nb is added to the alloy, the formation of Nb_2_O_5_ in the oxide layer is detrimental to the oxidation resistance of the alloy [23]. This is because when Nb is oxidized at high temperatures, accompanied by the initial formation of the oxide film, oxygen diffuses inwards through the surface oxide film, and the Nb_2_O_5_ formed has a large PBR (Pilling–Bedworth ratio) value, which generates stress at the oxide film–metal interface, leading to cracking of the oxide film, and the oxidation kinetics changes from a parabolic law to a linear law [24]. It has also been found that the addition of excessive Nb to the Fe-25Ni-10Cr-4.5Al alloy results in the formation of an Fe_2_Nb phase, which hinders the outward diffusion of Al atoms within the matrix, negatively affecting the formation of Al_2_O_3_ [25]. In conclusion, the effect of the Nb element on Al_2_O_3_ formation is not well explained and requires further investigation.

In addition, the lower oxygen pressure is conducive to reducing the oxygen activity and its permeability, ultimately promoting the selective external oxidation of Al_2_O_3_. In this paper, the alloy composition is designed as Fe-35Ni-20Cr-xAl-yNb (x = 0, 2, 4, 6 wt.%; y = 0, 1, 2 wt.%), and the effects on the oxidation behavior and products of the alloy are investigated by varying the contents of Al and Nb as well as the oxygen pressure, and using the thermodynamic calculations to explain the oxidation mechanism of the Nb element which promotes Al_2_O_3_ formation. This research offers further references for AFA alloy research.

## 2. Experimental Materials and Methods

### 2.1. Materials

Pre-oxidation experiments on Fe-35Ni-20Cr-xAl-yNb (x = 0, 2, 4, 6 wt.%; y = 0, 1, 2 wt.%) alloys were carried out using Fe, Ni, Cr, Al and Nb metal particles of 99.99% purity (Beijing Licheng Innovation Metal Materials Technology Co., Ltd., Beijing, China), with a total mass of 15 g of the alloy to be melted according to the ratio of the components, using a non-consumable vacuum arc furnace of type WK-ll. The melted samples were polished to remove the oxide layer on the surface; encapsulated in vacuum quartz tubes; and annealed at 1000 °C for more than 72 h to homogenize the organization and eliminate internal stresses. The samples were cut into thin slices of 10 mm × 8 mm × 2 mm and polished step by step with 400 mesh~2000 mesh SiC sandpaper and diamond polishing media. Before the oxidation test, the samples were cleaned with acetone and then distilled water, placed in ethanol and washed by ultrasonic shock for 2 min, and then removed and dried by cold air blowing. To ensure experimental accuracy and to prevent oxidation and moisture effects on the material, the samples were vacuum sealed and stored in a vacuum drying cabinet.

### 2.2. Pre-Oxidation Experiment

**Figure 1:**
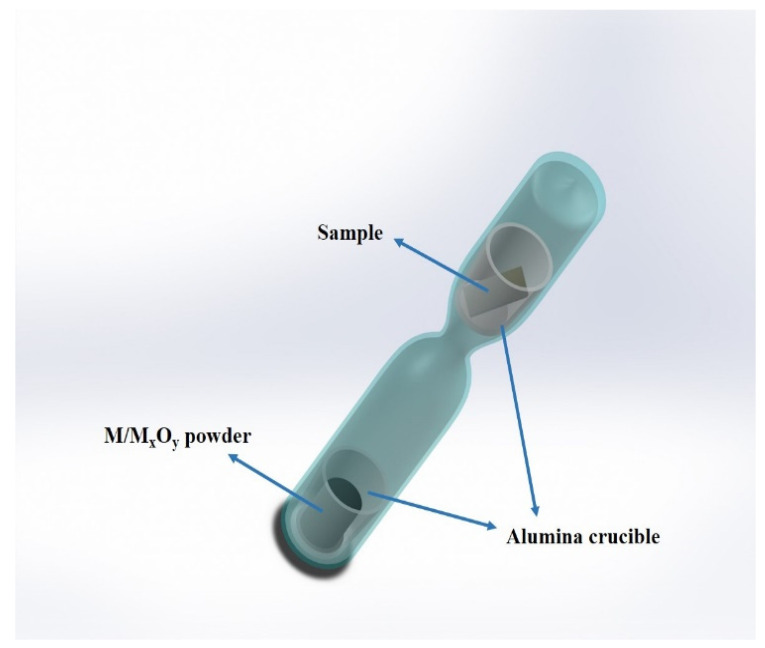
Oxidation device schematic.

Referring to the experiment of Ranganathan [26], Figure 1 shows the schematic diagram of the experimental setup of the embedding method. In this experiment, Fe/FeO powder and Cr/Cr_2_O_3_ powder with 99.99% purity and Cr/Cr_2_O_3_ powder were used in accordance with the atomic ratios of 1:1 and 2:1 and were prepared in batches of 2 g each, which were fully milled and mixed to form a block after compression. Then the powders and the alloy specimens were put into small crucibles, which were finally sealed into the ends of a vacuum quartz tube.

Heating the metal and its oxide powders for thirty minutes to attain initial equilibrium of oxygen partial pressure in the quartz tube was accomplished by employing a dual temperature tube furnace. The metal and oxide combination created a stable environment with low oxygen pressure (Table 1). The oxygen pressure and alloy oxidation temperature were regulated by adjusting temperatures at either end of the dual temperature tube furnace. Selective oxidation was conducted for a duration of 5 h at an oxidation temperature of 1000 °C because the Al_2_O_3_ in the oxidation product transformed from metastable oxide to stable α-Al_2_O_3_ at this high temperature (1000 °C and over).

### 2.3. Sample Analysis

Scanning electron microscope (SEM, JSM-6510, Tokyo, Japan) with energy dispersive spectroscopy (EDS, INCA, Oxford, UK) was utilized to examine the morphology, chemical composition and elemental distribution of the surface and cross-section of the oxide film. The surface morphology image of the oxidized sample was SEI, while BEI was used to analyze the chemical composition of the sample cross-section. The composition of the oxides was determined by X-ray diffraction (XRD, D/MAX 2500PC, Tokyo, Japan) with Cu Kα radiation at 40 kV and 100 mA.

The oxidation phase diagram and the equilibrium composition of the oxide under different oxidation conditions for the Fe–Ni–Cr–Al–Nb system were calculated using the iron-based database FeStel and the oxide database FToxid in FactSage 8.3 [27]. The influence of the Nb element content variation on the activity of other elements in the Fe–Ni–Cr–Al–Nb system was calculated using Pandat 2022 [28].

## 3. Results and Discussion

### 3.1. Effect of Al on the Pre-Oxidation Behavior of Fe-35Ni-20Cr-xAl Alloy

#### 3.1.1. The Microstructure of the Fe-35Ni-20Cr-xAl Alloy

**Figure 2:**
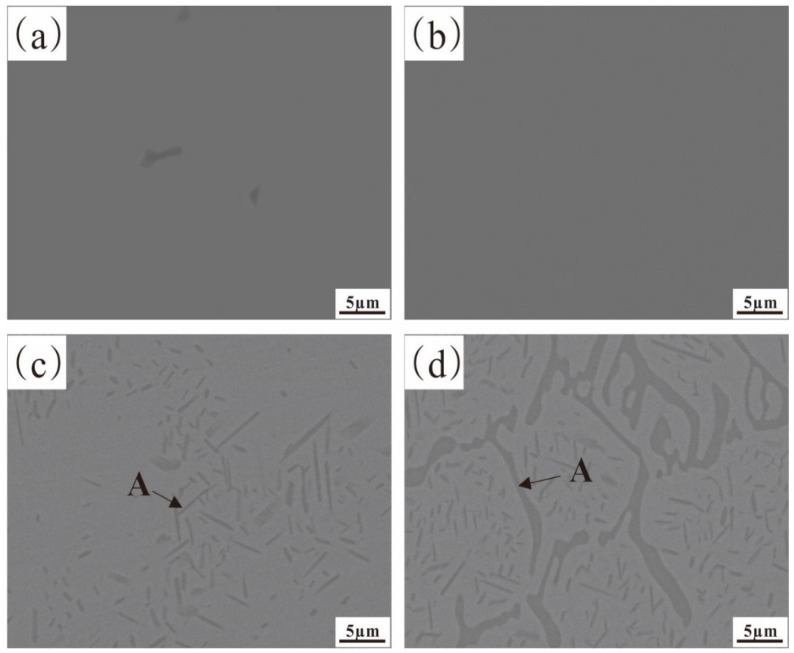
The microstructure of Fe-35Ni-20Cr-xAl alloys after annealing: (**a**) x = 0; (**b**) x = 2; (**c**) x = 4; and (**d**) x = 6.

**Figure 3:**
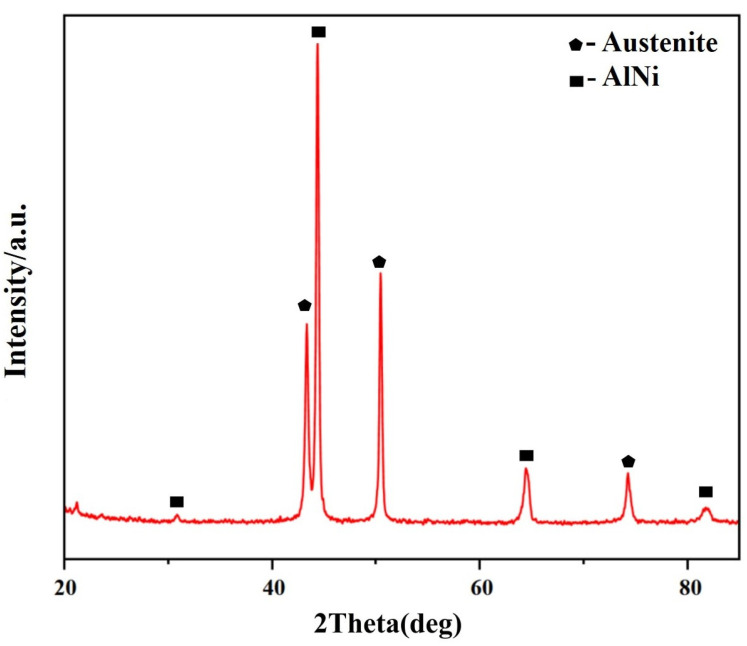
The XRD spectrum of Fe-35Ni-20Cr-6Al alloy after annealing.

Figure 2 and Figure 3, respectively, show the original microstructure of Fe-35Ni-20Cr-xAl (x = 0, 2, 4, 6) alloys with different Al contents after annealing at 1000 °C for 72 h, and the XRD patterns of the Fe-35Ni-20Cr-6Al alloy.

This shows that the alloys with 0 wt.% and 2 wt.% Al additions exhibit a homogeneous matrix (Figure 2a,b). With increasing Al content, the alloys with 4 wt.% and 6 wt.% Al additions show a two-phase region in the matrix, consisting of a gray austenite phase and a precipitate phase A. EDS analysis revealed that the composition of the precipitate phase A (at.%) was Al: 40.1%, Cr: 5.9%, Fe: 9.4%, and Ni: 44.6%, with an approximate Al to Ni atomic ratio of 1:1. The XRD in Figure 3 shows that the precipitated phase A is an AlNi phase, which has a high peak when Al content is added up to 6 wt.%.

#### 3.1.2. Calculation of Oxidation Phase Diagram

**Figure 4:**
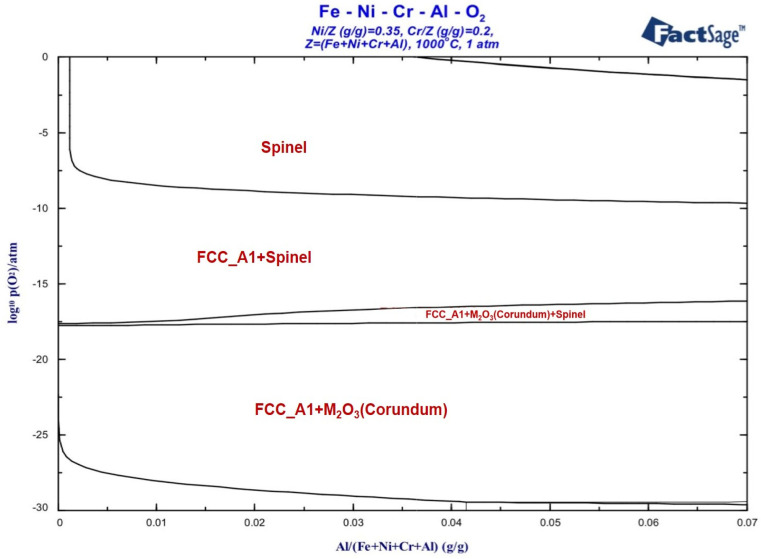
Oxidation phase diagram of Fe-35Ni-20Cr-xAl alloys at 1000 °C.

By calculating the oxidation phase diagram of the alloy, the appropriate oxidation conditions were selected according to the oxidation products so as to obtain a stable oxide film. As shown in Figure 4, the oxidation phase diagrams of the Fe-35Ni-20Cr-xAl alloy system with different oxygen pressures (y-axis) and Al content variations (x-axis) at 1000 °C were calculated by using Factsage, and the alloy compositions were designed to be the same as those of the experimental samples. From the plots, the main products of oxidation were oxides M_2_O_3_ and spinel, the main composition of oxides M_2_O_3_ is Cr_2_O_3_ and Al_2_O_3_, and the main composition of spinel is FeCr_2_O_4_.

As the oxygen pressure decreases, the oxidation products transform gradually from spinel to the M_2_O_3_ (Figure 4). As the pressure drops below 10^−18^ atm, the spinel phase disappears and only the stable M_2_O_3_ phase exists. Between 10^−17^ atm. and 10^−18^ atm., the curve of transformation (spinel to M_2_O_3_) increases slightly with increasing aluminum content. The spinel region above the curve decreases. This indicates that the increase in Al content promotes the formation of M_2_O_3_ and hinders the formation of spinel. The stability region of spinel and M_2_O_3_ is broadened with the increase in Al content. To investigate the effect of different Al contents on the conversion of oxidation products, the experimental oxygen pressure range had to be set at 10^−17^ atm. and below.

#### 3.1.3. Analysis of Pre-Oxidation Products

**Figure 5:**
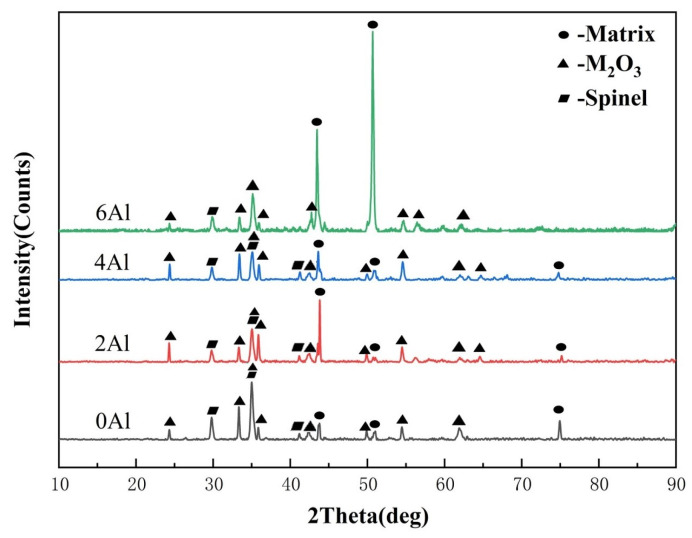
The XRD of Fe-35Ni-20Cr-xAl (x = 0, 2, 4, 6 wt.%) alloys at 1000 °C under oxygen pressure of 10^−17^ atm. After pre-oxidation for 5 h.

Figure 5 shows the XRD patterns of oxides of the Fe-35Ni-20Cr-xAl alloy (x = 0, 2, 4, 6) at 1000 °C under oxygen pressure of 10^−17^ atm. after pre-oxidation for 5 h. From the XRD pattern and the analysis of the chemical composition of the surface oxides in Table 2, it was found that the main oxidation products of the alloy without Al addition were Cr_2_O_3_ and spinel. The spinel component was mostly comprised of FeCr_2_O_4_. In the XRD patterns of the Al-added alloy, additional peaks can be observed on the diffraction peaks of the original M_2_O_3_, mainly generated by the promotional effect of the Al on the formation of M_2_O_3_. Furthermore, the XRD patterns of the alloy, which contains 6 wt.% Al after pre-oxidation, exhibit robust matrix peaks. This could be due to the occurrence of peeling of the oxidation products, exposing the alloy matrix in the case of the Fe-35Ni-20Cr-6Al alloy.

**Figure 6:**
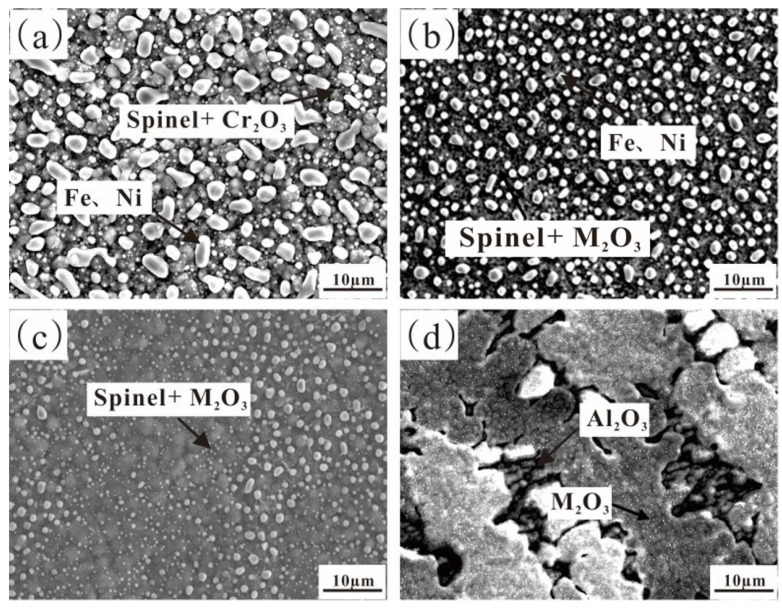
Surface SEM images of Fe-35Ni-20Cr-xAl alloys at 1000 °C under oxygen pressure of 10^−17^ atm. after pre-oxidation for 5 h: (**a**) x = 0; (**b**) x = 2; (**c**) x = 4; and (**d**) x = 6.

Figure 6 and Table 2, respectively, show the surface oxide morphology SEM images and their chemical compositions of the alloy after pre-oxidation for 5 h at 1000 °C under oxygen pressure of 10^−17^ atm. The XRD analysis in Figure 5 supports the observation that a composite oxide film consisting of spinel FeCr_2_O_4_ and M_2_O_3_ (Cr_2_O_3_ in this instance) was formed on the surface of the undoped Al alloy. Many iron and nickel particles adhered to the oxide film due to compressive stress [24]. At this point, high temperature caused an increase in the volume of internal oxides, resulting in compressive stress. The solvent components of nickel and iron were expelled to relieve the stress.

Furthermore, the oxide film was observed to have an uneven and loosely connected surface (Figure 6a). It was observed in Figure 6b–d that the diffused Fe and Ni particles on the alloys with addition of Al became smaller and significantly reduced in number, while the coverage area of surface oxide increased and gradually became smoother. At 2 wt.% Al content, the Cr content in the surface oxide layer increased while the Fe content decreased. This indicates a decrease in the amount of spinel (FeCr_2_O_4_) in the oxide, but the additional increase in Cr content indicates an increase in M_2_O_3_ suggesting that the addition of Al inhibited the formation of spinel. At 4 wt.% Al content, EDS compositional analysis demonstrated a notable decrease in Fe content in the oxide, primarily composed of the Cr element but with a small amount of Al present. This indicates that a small quantity of Al_2_O_3_ was solid-solved in the Cr-rich M_2_O_3_. When the Al content rose to 6%, large and continuous M_2_O_3_ oxide layers were observed without any Fe and Ni particle diffusion. Many Al_2_O_3_ particles materialized at M_2_O_3_ interblock gaps, revealing that the surface oxide had split and peeled off, uncovering the underlying Al_2_O_3_. This observation corresponds with the detection of diffraction peaks from exposed Al_2_O_3_ in the XRD patterns. The weak bond between the oxide film and the substrate is consistent with the speculation made beforehand.

**Figure 7:**
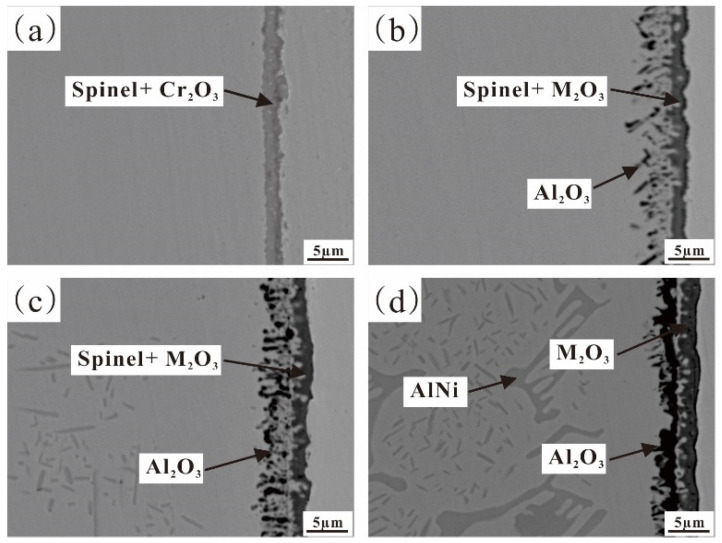
Cross-sectional SEM images of Fe-35Ni-20Cr-xAl alloys at 1000 °C under oxygen pressure of 10^−17^ atm. after pre-oxidation for 5 h: (**a**) x = 0; (**b**) x = 2; (**c**) x = 4; and (**d**) x = 6.

Figure 7 and Table 3, respectively, show the cross-sectional SEM images and chemical compositions of Fe-35Ni-20Cr-xAl (x = 0, 2, 4, 6) alloys pre-oxidized for 5 h at 1000 °C under oxygen pressure of 10^−17^ atm. The alloy containing the Al element demonstrated an additional inner layer of Al_2_O_3_ film when contrasted with the undoped Al alloy. The composite oxide film formed on the surface of the alloy with 2% Al addition had a thickness of about 1.5 μm. The inner layer of Al_2_O_3_ was irregularly distributed and had a depth of about 4–5 μm, which was discontinuous. When the Al content reached 4%, the thickness of the oxide layer became deeper and the amount of inner oxide increased significantly. The inner layer of Al_2_O_3_ gradually accumulated and became continuous. According to Figure 7c, it can be observed that the AlNi phase near the oxide layer in the matrix decreases, indicating that the Al element in the inner oxide Al_2_O_3_ mainly comes from the AlNi phase, which is consistent with previous studies [25]. In addition, there was an unoxidized matrix between the oxide layers.

**Figure 8:**
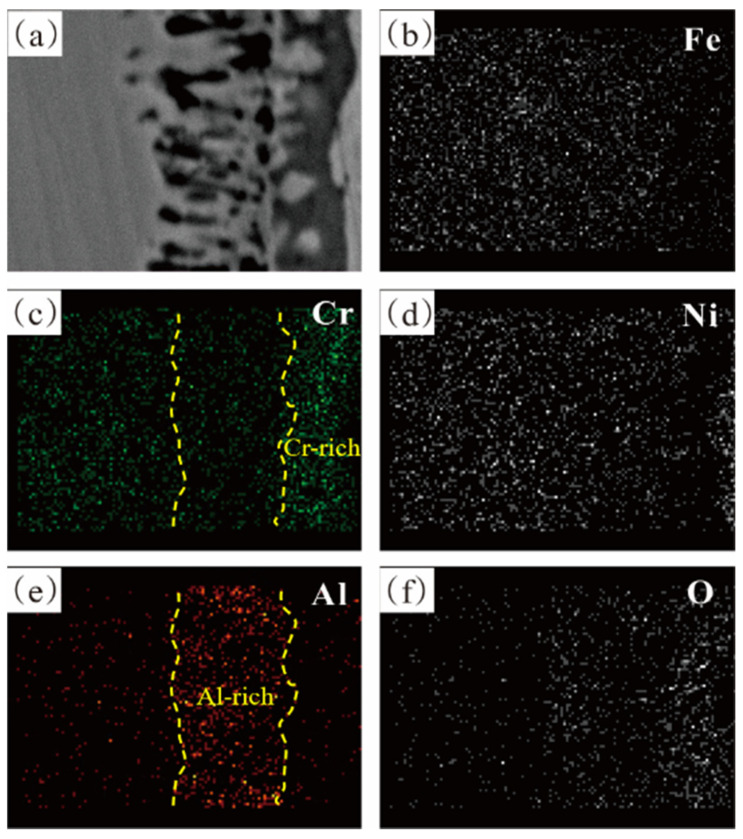
Cross-sectional scanning element distribution mapping of Fe-35Ni-20Cr-4Al alloy at 1000 °C under oxygen pressure of 10^−17^ atm. after pre-oxidation for 5 h.

Figure 8 displays the element distribution of the alloy containing 4% Al, which confirms that the oxide film consists of a Cr-rich M_2_O_3_ oxide outer layer and an Al_2_O_3_ inner layer. As the Al content increased to 6 wt.%, Al atoms diffused and accumulated towards the surface, the internal Al_2_O_3_ becoming narrower and more continuous, effectively blocking inward diffusion of oxygen atoms. The alloy underwent further oxidation, but severe internal oxidation occurred in the subsurface, increasing the PBR value which is an important criterion for judging the integrity of the oxide film [29,30]. The compressive stress in the oxide layer increased, making the outer M_2_O_3_ unstable and leading to partial peeling of the oxide film, as shown in Figure 6d. In addition, when the Al content reached 6 wt.%, the AlNi phase inside the matrix was significantly more than that in the 4% Al alloy, as shown in Figure 2. In conclusion, the 4% Al alloy had better oxide film integrity than the 6% Al alloy where surface peeling occured.

**Figure 9:**
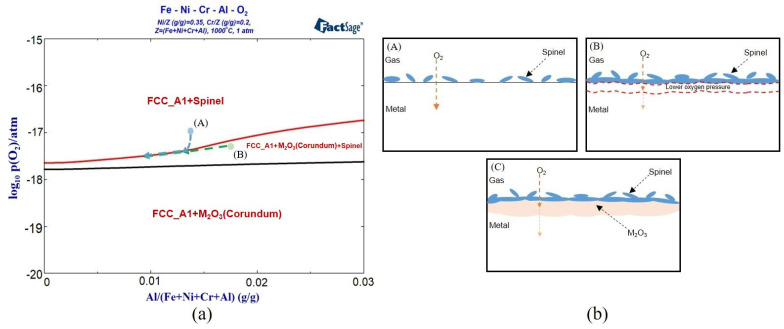
Schematic diagram of the dynamic oxidation process: (**a**) variation of surface composition in the alloy; and (**b**) (A–C) is a schematic diagram of the formation process of secondary oxides.

It is worth noting that the experimental results also revealed that after oxidation of alloys with 0 wt.% and 2 wt.% Al content at 1000 °C and under oxygen pressure of 10^−17^ atm., both spinel and M_2_O_3_ phases appeared. Figure 4 shows that only spinel was present in the oxidation products of the addition of 0 wt.% and 2 wt.% Al alloys under the same oxidation conditions. This was attributed to the fact that the oxidation process is a kinetic process, where the depletion of elements in the matrix and changes in oxygen pressure affect the oxidation results. The oxidation phase diagram in Figure 4 represents a stable phase diagram for primary oxidation. It provides valuable information for the secondary oxidation process and oxidation sequence of the alloy [31]. Firstly, spinel was formed on the surface of alloys with 0–2 wt.% Al addition at 1000 °C and under oxygen pressure of 10^−17^ atm. Spinels on the surface of the alloy impeded the diffusion of oxygen into the interior of the matrix, leading to a decrease in the oxygen pressure between the spinels and the matrix. The reduction in oxygen pressure promoted the formation of M_2_O_3_, which led to depletion of matrix elements due to oxidation. As shown in Figure 9a, after a period of time from the start of the oxidation reaction, both points A and B in the figure shifted to a location with a lower oxygen pressure and Al content. After secondary oxidation forming spinel and M_2_O_3_, the oxidation reaction reached equilibrium. The specific oxidation behavior is shown in Figure 9b.

**Figure 10:**
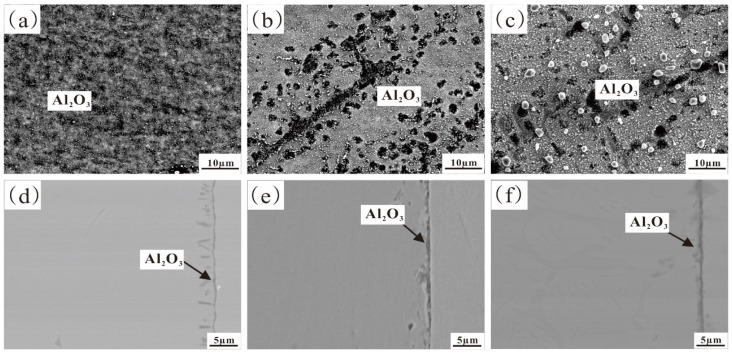
Surface and cross-sectional SEM images of Fe-35Ni-20Cr-xAl alloys at 1000 °C under oxygen pressure of 10^−25^ atm. after pre-oxidation for 5 h: (**a**,**d**) x = 2; (**b**,**e**) x = 4; and (**c**,**f**) x = 6.

Figure 10 shows the SEM images of the surface and cross-section of Fe-35Ni-20Cr-xAl (x = 0, 2, 4, 6) alloys after pre-oxidation at 1000 °C under oxygen pressure of 10^−25^ atm. for 5 h. It was found that a thin layer of Al_2_O_3_ film forms on the outer surface of the alloy containing 2% Al, and internal oxidation of Al_2_O_3_ occurs in the subsurface with a depth of about 2–3 μm. Although the Cr content was much higher than the aluminum Al content, and the diffusion rate of Cr was faster [32], Cr was difficult to oxidize under low oxygen pressure because of there being no thermodynamic driving force. Only a thin and discontinuous layer of Al_2_O_3_ formed on the surface, which was unable to block the inward diffusion of oxygen atoms. Consequently, Al_2_O_3_ particles were formed in the subsurface of the alloy. When the Al content increased to 4%, a more prominent and thicker Al_2_O_3_ layer formation occurred on the outer surface and the granular Al_2_O_3_ in the interior disappeared. Nevertheless, the oxide film remained porous and not dense, as shown in Figure 10b,e. When the aluminum content was increased to 6%, the Al_2_O_3_ layer became thicker and a considerable number of Fe and Ni particles were dispersed to the surface of the alloy, resulting in fewer surface defects and holes. The formation of these holes was predominantly attributed to the diffusion of Fe and Ni particles from the alloy’s interior to its surface. Compared to the oxide film developed on the alloy surface under an oxygen pressure of 10^−17^ atm., the composite oxide film formed on the same surface under an oxygen pressure of 10^−25^ atm. was fully transformed into a protective Al_2_O_3_ layer. These results indicated that decreasing the oxygen pressure facilitates the creation of external Al_2_O_3_.

### 3.2. The Influence of Nb on the Pre-Oxidation Behavior of Fe-35Ni-20Cr-4Al-yNb Alloy

#### 3.2.1. The Microstructure of the Fe-35Ni-20Cr-4Al-yNb Alloy

**Figure 11:**
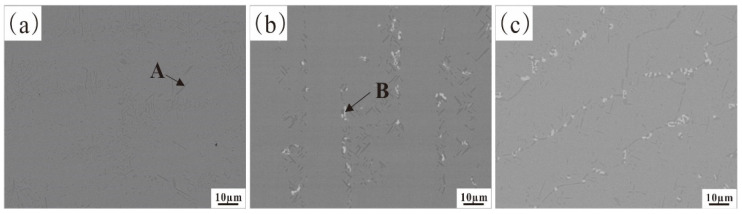
The microstructure of Fe-35Ni-20Cr-4Al-yNb alloys after annealing: (**a**) y = 0; (**b**) y = 1; and (**c**) y = 2.

**Figure 12:**
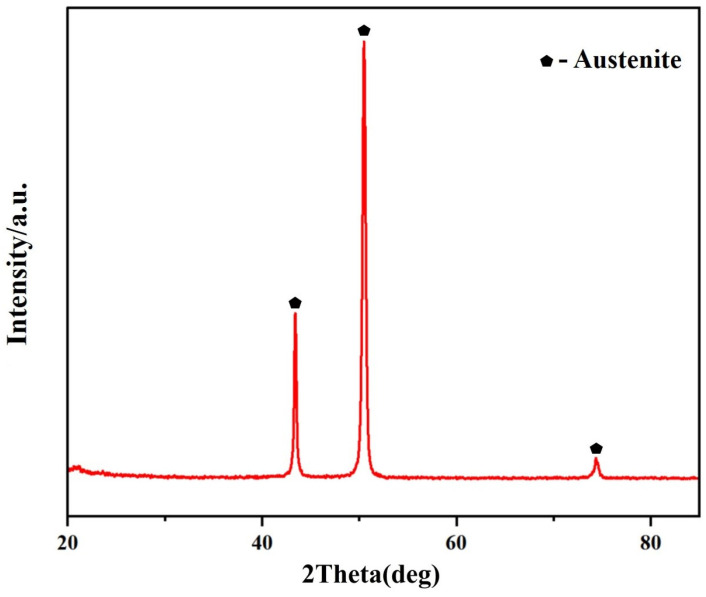
The XRD spectrum of Fe-35Ni-20Cr-4Al-2Nb alloy after annealing.

After considering the variations in Al content in the alloy, the one with 4% Al was chosen as the ideal option for analyzing the impact of Nb content. Figure 11 shows the microstructures of Fe-35Ni-20Cr-4Al-yNb (y = 0, 1, 2) alloys with different Nb contents after annealing at 1000 °C for 72 h. From this, it can be observed that in the original alloy without addition of Nb, there are two phases present: a gray matrix phase (FCC phase) and a precipitate phase A (AlNi phase). After the addition of niobium to the alloy, some white phases B (Nb-rich phase) were observed near the grain boundaries. With increase in Nb content, the Nb-rich phase became more abundant and denser. Upon analysis by EDS, the composition of the Nb-rich phase was as follows (in %): Al: 2.2%; Cr: 10.6%; Fe: 32.6%; Nb: 28.5%; Ni: 26.1%. The XRD pattern in Figure 12 shows that only peaks of the austenite phase appeared. In addition to the matrix phase, there were also metal compound phases of Ni–Nb and Ni–Al–Nb in the austenite phase.

#### 3.2.2. Calculation of Oxidation Phase Diagram

**Figure 13:**
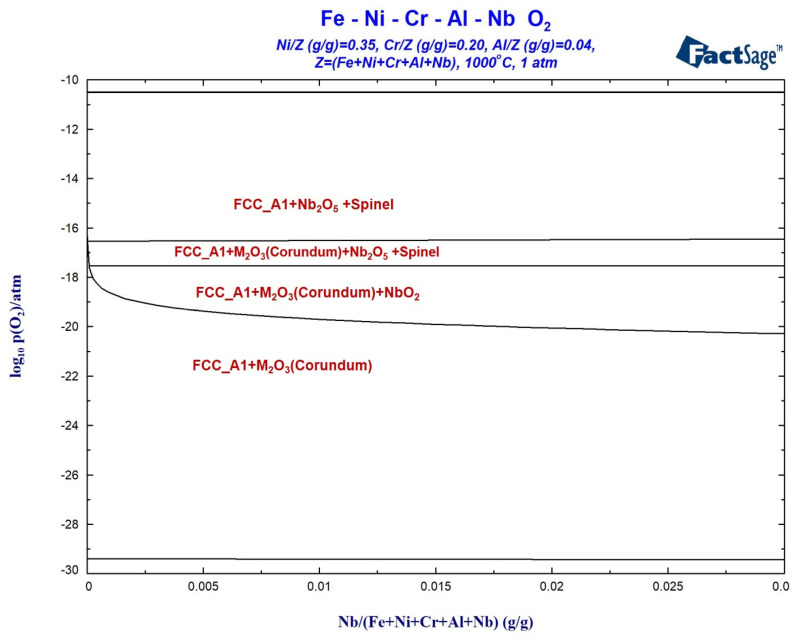
Oxidation phase diagram of Fe-35Ni-20Cr-4Al-yNb alloys at 1000 °C.

Using the same calculation method as depicted in Figure 4, we calculated the oxidation phase diagram of the Fe-35Ni-20Cr-4Al-yNb alloy system at different oxygen pressures and Nb concentrations at 1000 °C. The results are presented in Figure 13, which demonstrates the formation of M_2_O_3_, spinel, and small amounts of Nb oxide. If the oxygen pressure exceeds or is close to 10^−17^ atm., Nb_2_O_5_ will form; however, at lower oxygen pressures, NbO_2_ is the product. As the amount of Nb added increases, the limiting pressure required to form NbO_2_ decreases. When the oxygen pressure is below 10^−20^ atm., the only present oxide type is M_2_O_3_.

**Figure 14:**
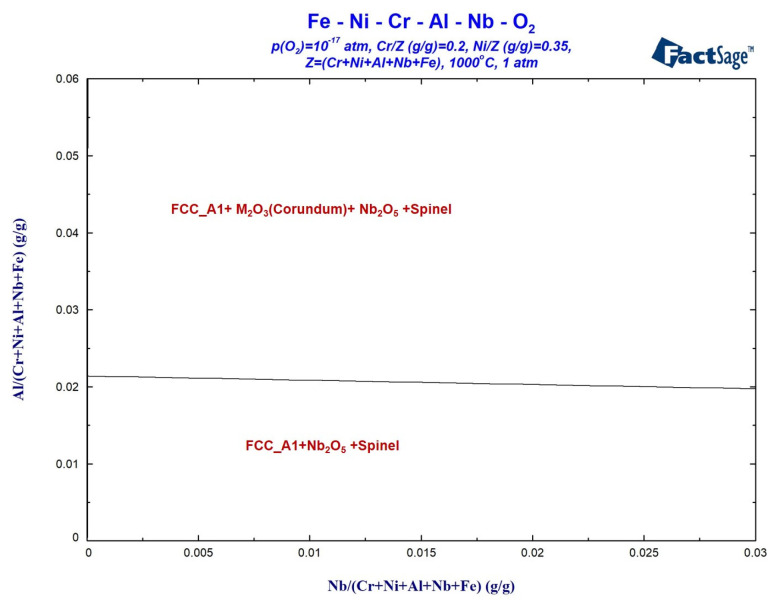
Oxidation phase diagram of Fe-35Ni-20Cr-xAl-yNb alloys at 1000 °C under oxygen pressure of 10^−17^ atm.

Figure 14 shows the calculated oxidation phase diagram of the Fe-35Ni-20Cr-xAl-yNb system at 1000 °C under the oxygen pressure of 10^−17^ atm. with varying levels of Nb and Al present. The x-axis represents the Nb content range of 0–0.03, and the y-axis represents the Al content range of 0–0.06. It is evident that at this oxygen pressure, oxidation reactions generate spinel and Nb_2_O_5_ when Al content is below approximately 2%. As the Al content increases, spinel disappears and M_2_O_3_ is formed. This oxygen pressure is close to the critical pressure for oxide formation, which makes studying the oxidation products at this pressure beneficial for explaining the selective oxidation behavior of the alloy. Figure 14 shows a decreasing trend in the plotted curve at oxygen pressures of 10^−17^ atm. As the Nb content increases, it has a third element effect, leading to a decrease in the critical Al content required for the formation of M_2_O_3_. The calculated result indicates that the addition of Nb promotes the formation of M_2_O_3_ and hinders the formation of spinel.

#### 3.2.3. Analysis of Pre-Oxidation Products

**Figure 15:**
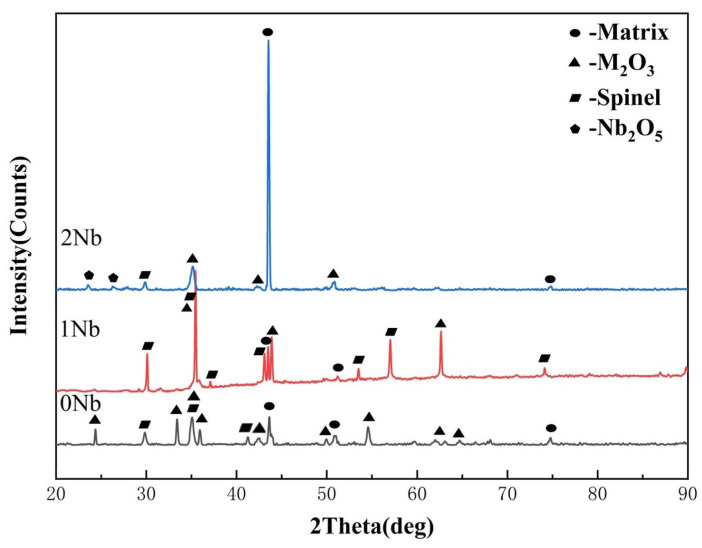
The XRD of Fe-35Ni-20Cr-4Al-yNb (y = 0, 1, 2wt.%) alloys at 1000 °C under oxygen pressure of 10^−17^ atm. after pre-oxidation for 5 h.

**Figure 16:**
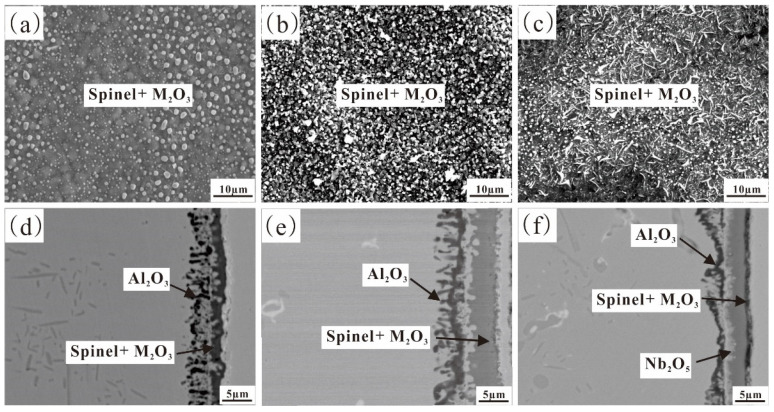
Surface and cross-sectional SEM images of Fe-35Ni-20Cr-4Al-yNb alloys at 1000 °C under oxygen pressure of 10^−17^ atm. after pre-oxidation for 5 h: (**a**,**d**) x = 0; (**b**,**e**) x = 1; and (**c**,**f**) x = 2.

Figure 15 and Figure 16 show the XRD patterns, surface morphologies, and sectional morphologies of Fe-35Ni-20Cr-4Al-yNb (y = 0, 1, 2) alloys after pre-oxidation at 1000 °C under oxygen pressure of 10^−17^ atm. for 5 h. Chemical compositions (at.%) for each phase in Figure 16d–f are presented in Table 4. The XRD analysis shows that the oxide M_2_O_3_ (M: Al and Cr) and a small amount of spinel served as the main oxidation products on the alloy surface without Nb addition. When 1% Nb was added, the thickness of the oxide layer on the alloy surface increased. The XRD analysis still displayed peaks of spinel FeCr_2_O_4_ and M_2_O_3_ as the oxide phases on the alloy surface; however, no peak for Nb_2_O_5_ was observed. This was primarily due to the small quantity of Nb added and the negligible content of Nb_2_O_5_ compared to other oxide products. As the Nb content increased to 2%, the spinel peak showed a significant decrease, and the XRD pattern mainly displayed the matrix peak. This related to the detachment of the oxide surface layer. The PBR value of Nb_2_O_5_ is 2.68 [33], resulting in significant compressive stress. Excessive addition of Nb could cause the cracking of the oxide film, which is consistent with previous studies [24]. To avoid high levels of Nb, which can lead to flaking of the oxide film, the Nb content in this study was controlled at 2 wt.% and below. It could be observed from the sectional image that Nb_2_O_5_ was tightly adhered to the metal surface underneath the outer oxide layer.

Figure 16a shows that the oxide on the surface of the alloy without addition of Nb is mainly M_2_O_3_ and spinel. In addition, observation of the cross-sections of the samples reveals the presence of an internal layer of 4–5 μm discontinuous dendritic Al_2_O_3_ (Figure 16d). Figure 16b and the EDS evidence analysis in Table 4 revealed the presence of irregular-shaped spinel particles on the 1% Nb addition alloy surface. Compared to the Nb-free alloy, the addition of Nb resulted in a significant reduction in Fe and Ni elements present in the surface oxide, indicating a decrease in spinel content. The cross-section observation revealed a significant thickening of the external oxide layer, with a thickness of about 4–5 μm. The internal Al_2_O_3_ became more abundant and continuous, indicating that the addition of Nb promoted the formation of M_2_O_3_, which was consistent with the calculation results. There remained an area that was not oxidized between the inner and outer oxide layers. Upon further increasing the Nb content in the alloy to 2%, it was found that the inner Al_2_O_3_ content increased further. Spinel was still present on the surface of the alloy; however, the concentration of Fe and Ni in the surface consistently decreased. This suggests that the addition of Nb helped to hinder the formation of spinel. Figure 16d–f illustrates that both the internal and external oxide layers became more condensed and continuous, while the unoxidized area in the middle layer diminished with an increase in Nb content. The addition of Nb enhanced the development of Al_2_O_3_ and M_2_O_3_, thus improving the alloy’s capacity for oxidation resistance. Additionally, a region rich in Nb_2_O_5_ was observed.

**Figure 17:**
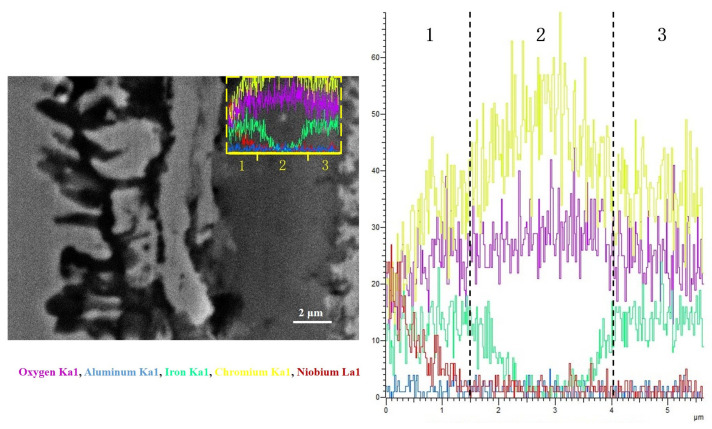
The line scanning image of the outer oxide layer of Fe-35Ni-20Cr-4Al-1Nb alloy at 1000 °C under oxygen pressure of 10^−17^ atm. after pre-oxidation for 5 h.

Figure 17 shows the line scan image of the outer oxide layer of Fe-35Ni-20Cr-4Al-1Nb alloy after pre-oxidation for 5 h at 1000 °C under oxygen pressure of 10^−17^ atm. From the Figure, this outer layer can be divided into three parts. It was found that in the outer spinel + M_2_O_3_ oxide layer, the Fe element content decreased from the outer to the inner region, while the Cr element content increased. This indicated a transition from spinel to M_2_O_3_ in the oxide layer from the outer to the inner region. When moving further inwards, the Fe content increased again, as test position 1 is close to the unoxidized substrate. The oxide layer formed on the alloy includes an outermost layer containing a mixture of spinel + M_2_O_3_, an innermost dark Al_2_O_3_ layer and an intermediate matrix. The Nb element content gradually increased from the outer to the inner region, confirming the formation and position of Nb_2_O_5_.

**Figure 18:**
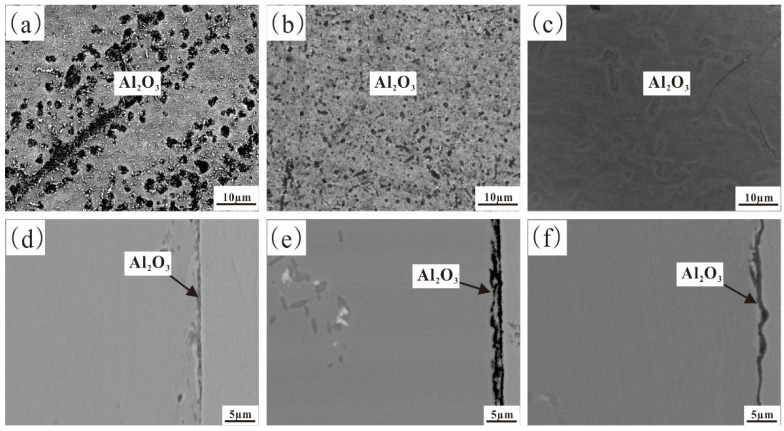
Surface and cross-sectional SEM images of Fe-35Ni-20Cr-4Al-yNb alloys at 1000 °C under oxygen pressure of 10^−25^ atm. after pre-oxidation for 5 h: (**a**,**d**) y = 0; (**b**,**e**) y = 1; and (**c**,**f**) y = 2.

Figure 18 shows the surface and cross-sectional SEM images of Fe-35Ni-20Cr-4Al-yNb (y = 0, 1, 2) alloys after pre-oxidation for 5 h at 1000 °C under oxygen pressure of 10^−25^ atm. In the alloy without addition of Nb, the main oxide on the surface was Al_2_O_3_ (Figure 18a,d). The surface oxide film was porous and accompanied by outward diffusion of Fe and Ni particles, indicating that the oxide film was extremely thin and porous, making it difficult to impede the outward diffusion of alloy elements. When the alloy was doped with 1% Nb, the surface Al_2_O_3_ layer slightly increased in density as Fe and Ni particles ceased to diffuse to the surface. The addition of 2% Nb caused the oxide on the alloy surface to become both dense and continuous, without any voids or defects, which was confirmed via the cross-sectional image (Figure 18c,f). Under oxygen pressure of 10^−25^ atm., neither an outer oxidized M_2_O_3_ nor spinel on the alloy surface existed. The inclusion of Nb resulted in the densification and continuity of the Al_2_O_3_ surface film.

### 3.3. Thermodynamic Calculation and Analysis

The experiment took place at an oxidation temperature of 1000 °C. The oxidation reaction of metal M can be depicted by the following equation:

(1) $$M+O_{2}⟺MO_{2}$$

The Gibbs free energy of the metal oxidation reaction is typically calculated using the Van’t Hoff isothermal formula:

(2) $$\Delta G=\Delta G^{\Theta }+\mathrm{RT}\ln K$$

where $\Delta G^{\Theta }$ represents the change in free energy when all substances are in their standard states, R is the gas constant, T is the absolute temperature, and $K=\frac{a_{{MO}_{2}}}{a_{M}\;\cdot a_{O_{2}}}$ is the equilibrium constant. Here, a represents thermodynamic activity, which reflects the deviation of a group of elements from the standard state. For a certain element i in gas, its activity can be expressed as $a_{i}=\frac{P_{i}}{P_{i}^{\Theta }}$, where $P_{i}$ is the vapor pressure of a condensed-phase component or the partial pressure of a gas-phase component, and $P_{i}^{\Theta }$ is its corresponding value at the standard state. Under high temperature and low pressure, gases can be approximated as ideal gases, so $a_{O_{2}}=P_{O_{2}}$. The progress of the oxidation reaction is affected by the activity of oxygen, metal and its oxides, as specified in the reaction equation.

To clarify the relationship between the standard Gibbs free energy of oxide formation $\Delta G^{\Theta }$ and temperature, and compare the stability of oxides, the following relationships of the standard Gibbs free energy $\Delta G^{\Theta }$ of relevant oxides in this experiment at 1000 °C can be established: ${\Delta G}_{NiO}^{\Theta }>{\Delta G}_{FeO}^{\Theta }>{\Delta G}_{{Cr}_{2/3}O}^{\Theta }>{\Delta G}_{{Nb}_{2/5}O}^{\Theta }>{\Delta G}_{{Al}_{2/3}O}^{\Theta }$. Aluminum exhibits the highest affinity towards oxygen, and the oxide Al_2_O_3_ is considered the most stable. These findings are congruent with both experimental and computational results.

Furthermore, if the activities of M and $MO_{2}$ are taken as 1 [24], then Equation (2) can be simplified to:

(3) $$\Delta G=\Delta G^{\Theta }+\mathrm{RT}\ln\frac{a_{{MO}_{2}}}{a_{M}\cdot a_{O_{2}}}=\Delta G^{\Theta }-\mathrm{RT}\ln P_{O_{2}}$$

After the oxidation reaction reaches equilibrium, ΔG=0, the Equation (3) can be changed to:

(4) $$\Delta G^{\Theta }=\mathrm{RT}\ln P_{O_{2}}^{M/MO_{2}}$$

The equilibrium oxygen pressure of oxides can be expressed as:

(5) $$P_{O_{2}}^{M/MO_{2}}=exp\frac{\Delta G^{\Theta }}{\mathrm{RT}}$$

In the case of selective oxidation of alloys, the activities of the metal and oxide must be considered, and the equation is as follows:

(6) $$P_{O_{2}}^{eq}=\frac{a_{{MO}_{2}}}{a_{M}}exp\frac{\Delta G^{\Theta }}{\mathrm{RT}}=\frac{a_{{MO}_{2}}}{a_{M}}P_{O_{2}}^{M/MO_{2}}$$

Although at 1000 °C, ${\Delta G}_{{Cr}_{2/3}O}^{\Theta }>{\Delta G}_{{Nb}_{2/5}O}^{\Theta }>{\Delta G}_{{Al}_{2/3}O}^{\Theta }$, under oxygen pressure 10^−17^ atm., Cr diffused faster and its concentration was much higher than that of Al. Consequently, Cr was preferentially oxidized to form a Cr-rich M_2_O_3_, and Al_2_O_3_ formed in the subsurface region due to the weakening of oxygen pressure. Nb_2_O_5_ was formed between these two oxide layers. When the oxygen pressure was 10^−25^ atm., the oxygen activity decreased. Under this low oxygen pressure, only Al underwent oxidation, while Cr and Nb did not oxidize because of there being no thermodynamic driving force. This is consistent with the computational and experimental results.

**Figure 19:**
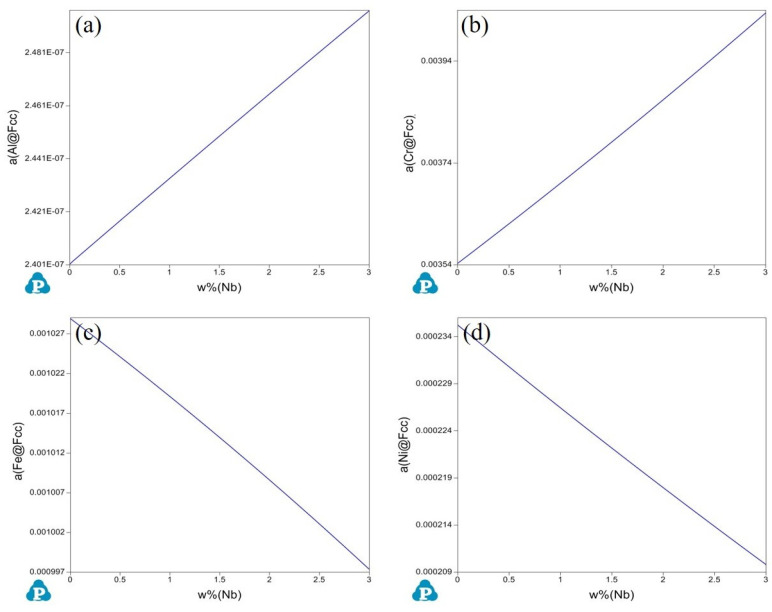
Effect of the Nb element on the activities of other elements in Fe-35Ni-20Cr-4Al-yNb alloys calculated by Pandat. (**a**) Activity of Al; (**b**) Activity of Cr; (**c**) Activity of Fe; (**d**) Activity of Ni.

The increase in activity of alloying elements promoted the formation of oxides. To explain the role of the Nb element, the influence of Nb on the activity of each element in the alloy was calculated using Panda, as shown in Figure 19. This shows that the addition of Nb increased the activities of Al and Cr by approximately 4% and 14%, respectively, which enhanced the driving force for the formation of Al_2_O_3_ and Cr_2_O_3_ in the alloy. Referring to existing research [34], it can be seen that the addition of the Nb element increased the diffusion ability of Cr and Al elements. The addition of Nb also reduced the activity of Fe and Ni, and weakened their diffusion ability. Additionally, with lower oxygen pressure, Fe and Ni reduced susceptibility to oxidation, which helped to inhibit the formation of the spinel phase produced by solid-state reactions.

**Figure 20:**
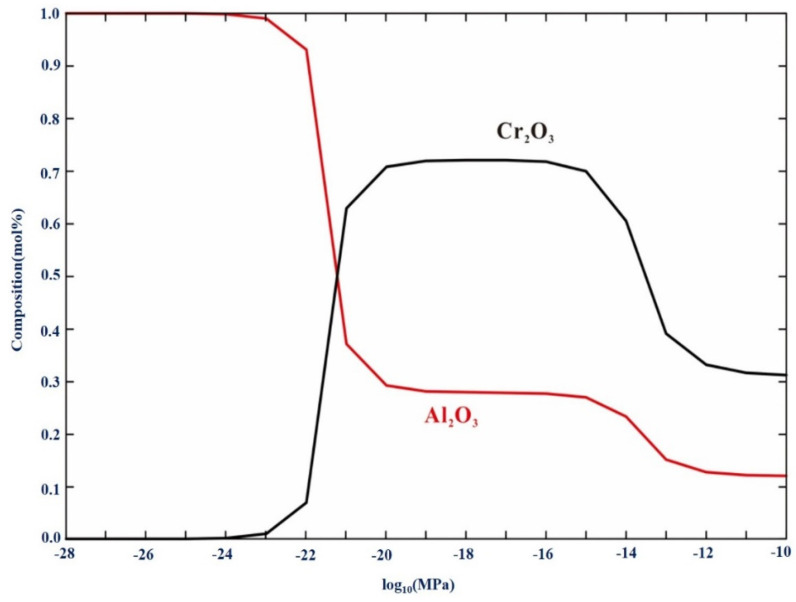
The equilibrium composition of oxide in Fe-35Ni-20Cr-4Al-2Nb alloy at 1000 °C under different oxygen pressures calculated by Factsage.

According to Equation (6), the influence of oxygen pressure on the driving force for oxide formation must be considered. The lower the oxygen pressure, the lower the driving force for oxide formation and the more difficult it is to form oxides. The composition of the oxide in the oxide layer of Fe-35Ni-20Cr-4Al-2Nb at 1000 °C under different oxygen pressures was calculated using Factsage, as shown in Figure 20. It was found that when the oxygen pressure is lower than 10^−17^ atm., the content of Al_2_O_3_ increases and the content of Cr_2_O_3_ decreases as the oxygen pressure decreases.

Combining Figure 9b and Figure 20, we find that even at the higher oxygen pressure of 10^−17^ atm., the Cr-rich M_2_O_3_ oxide formed on the surface hinders the diffusion of oxygen, and reduces oxygen activity. The concentration of oxygen decreased as it moves inward, resulting in lower oxygen activity. The order of oxide formation from inside to outside was Al_2_O_3_, Nb_2_O_5_, and Cr_2_O_3_, partly because of the following relationships ${\Delta G}_{{Cr}_{2/3}O}^{\Theta }>{\Delta G}_{{Nb}_{2/5}O}^{\Theta }>{\Delta G}_{{Al}_{2/3}O}^{\Theta }$.

As the oxidation process continued, a small amount of inner Al_2_O_3_ slowly grew and diffused outward. Thus, the outer layer formed M_2_O_3_ rich in Cr and contained a small amount of Al, which together with the underlying Al_2_O_3_ and Nb_2_O_5_, formed a protective film. When the level of oxygen pressure was below 10^−25^ atm., the excessively low pressure made it challenging for other elements to undergo oxidation. The extremely low oxygen activity resulted in only Al combining with oxygen on the alloy surface, creating a dense and continuous single layer of protective Al_2_O_3_ film.

## 4. Conclusions

The effects of Al and Nb additions on the low oxygen pre-oxidation of Fe-35Ni-20Cr-xAl-yNb (x = 0, 2, 4, 6 wt.%; y = 0, 1, 2 wt.%) alloys at 1000 °C under different oxygen pressures were studied. The oxidation mechanism of the alloy was analyzed by thermodynamic calculation and analysis, and the results are as follows:

(1) For Fe-35Ni-20Cr-xAl (x = 0, 2, 4, 6) alloys, at 1000 °C under oxygen pressures of 10^−17^ atm., the pre-oxidation protective film consisted of a continuous outer layer of FeCr_2_O_4_, M_2_O_3_ (M: Al and Cr), and an inner layer composite oxide film of Al_2_O_3_. Increasing the content of Al resulted in an increase in Al_2_O_3_ content in the oxidation products. Al promoted the formation of M_2_O_3_ and prevented the growth of FeCr_2_O_4_, leading to the formation of a continuous inner Al_2_O_3_ oxide film. When the Al content reached 6%, partial delamination of the oxide film occurred on the alloy surface.
(2) The addition of Nb enhanced the activity of Cr and Al elements while reducing the activity of Ni and Fe elements. Increase in element activity enhanced the driving force for the formation of their oxides. At an oxidation temperature of 1000 °C under oxygen pressure of 10^−17^ atm., the amount of M_2_O_3_ formed on the alloy surface increased with the addition of Nb, resulting in a gradually denser oxidation layer. More continuous internal oxidation of Al_2_O_3_ occurred, which inhibited the formation of spinel, and a Nb-rich oxide, Nb_2_O_5,_ appeared internally.
(3) As the oxygen pressure decreased, the driving force for oxide formation also decreased. The experiment and calculations show that the amount of Cr_2_O_3_ in the oxide layer decreased, while the amount of Al_2_O_3_ increased. When the oxygen pressure was as low as 10^−25^ atm., only Al_2_O_3_ was formed.
(4) By adding Nb and reducing the oxygen pressure, a continuous and dense Al_2_O_3_ oxide film on Fe-35Ni-20Cr-4Al-2Nb alloy pre-oxidized for 5 h at 1000 °C under oxygen pressure of 10^−25^ atm. was obtained.

## Figures and Tables

**Table 1:** Oxygen pressure provided by powder.

Powder	Temperature	P(O_2_)
Fe/FeO	860 °C	10^−17^ atm.
Cr/Cr_2_O_3_	900 °C	10^−25^ atm.

**Table 2:** Chemical composition of the oxidation product in Figure 6 (at.%).

Alloys	Phase	O	Al	Cr	Fe	Ni
Fe-35Ni-20Cr	Fe + Ni particles	8.7	--	4.6	32.6	54.0
	Spinel + Cr_2_O_3_	58.3	--	26.9	13.8	1.0
0Fe-35Ni-20Cr-2Al	Spinel + M_2_O_3_	56.2	0.8	30.0	11.9	1.1
Fe-35Ni-20Cr-4Al	Spinel + M_2_O_3_	60.7	1.1	30.5	6.4	1.3
Fe-35Ni-20Cr-6Al	M_2_O_3_	59.8	1.4	28.2	8.9	1.7
	Al_2_O_3_	59.2	36.5	2.2	1.6	0.5

**Table 3:** Chemical composition of the cross-sectional oxide layer in Figure 7 (at.%).

Alloys	Phase	O	Al	Cr	Fe	Ni
Fe-35Ni-20Cr	Spinel + Cr_2_O_3_	56.5	--	27.0	13.2	3.3
Fe-35Ni-20Cr-2Al	Spinel + M_2_O_3_	57.0	0.8	27.2	9.3	5.7
	Al_2_O_3_	42.1	21.7	5.6	17.1	13.5
Fe-35Ni-20Cr-4Al	Spinel + M_2_O_3_	57.4	0.5	28.0	8.7	5.4
	Al_2_O_3_	50.6	29.1	3.2	10.3	6.8
Fe-35Ni-20Cr-6Al	M_2_O_3_	60.6	1.6	24.0	11.4	2.4
	Al_2_O_3_	48.7	29.5	4.8	9.1	7.9

**Table 4:** Chemical composition of the cross-sectional oxide layer in Figure 15 (at.%).

Alloys	Phase	O	Al	Cr	Fe	Ni	Nb
Fe-35Ni-20Cr-4Al	Spinel + M_2_O_3_	57.4	0.5	28.0	8.7	5.4	--
	Al_2_O_3_	50.6	29.1	3.2	10.3	6.8	--
Fe-35Ni-20Cr-4Al-1Nb	Spinel + M_2_O_3_	61.1	0.3	30.8	5.1	2.6	0.1
	Al_2_O_3_	60.4	34.7	1.2	2.0	1.6	0.1
Fe-35Ni-20Cr-4Al-2Nb	Spinel + M_2_O_3_	64.6	0.6	30.8	2.3	1.4	0.3
	Al_2_O_3_	60.0	36.0	1.8	1.1	1.0	0.1

## Data Availability

Data are contained within the article.
